# Mechanical Stiffening Promotes Growth, Invasion-Associated Phenotypes, and Reduced Selumetinib Sensitivity in 3D Plexiform Neurofibroma Cultures

**DOI:** 10.3390/cells15100877

**Published:** 2026-05-12

**Authors:** Kyungmin Ji, Chenjun Shi, Jitao Zhang, Raymond R. Mattingly

**Affiliations:** 1 Department of Neurology, Henry Ford Health System, Detroit, MI 48202, USA; 2 Department of Pharmacology, Wayne State University, Detroit, MI 48201, USA; 3 Department of Physiology, Michigan State University, East Lansing, MI 48824, USA; 4 Department of Biomedical Engineering, Michigan State University, East Lansing, MI 48823, USA; 5 Department of Pharmacology and Toxicology, East Carolina University Brody Medical School, Greenville, NC 27834, USA

**Keywords:** plexiform neurofibroma, neurofibromatosis, extracellular matrix stiffness, mechanically induced drug resistance, lysyl oxidase, and p-glycoprotein

## Abstract

**Highlights:**

**What are the main findings?**

- Stiff extracellular matrix (ECM) promotes growth and invasion-associated phenotypes, including spread morphology and intracellular softening, in pNF1 tumor cells in 3D cultures.
- Stiff ECM is associated with reduced sensitivity to the MEK inhibitor selumetinib and upregulation of pathways associated with reduced drug response, including P-glycoprotein.

**What is the implication of the main finding?**

- ECM stiffness is a key regulator of pNF1 tumor behavior and therapeutic response beyond genetic drivers.
- Targeting ECM remodeling or mechanical properties may improve treatment outcomes in neurofibromatosis type 1.

**Abstract:**

Plexiform neurofibromas (pNF1s) are benign peripheral nerve sheath tumors caused by *NF1* loss, leading to dysregulated RAS/mitogen-activated protein kinase (MAPK) signaling. While the mitogen-activated protein kinase kinase (MEK) inhibitors, selumetinib and mirdametinib, can reduce tumor volume, surgical resection remains the primary treatment for immediate debulking and symptom relief. Complete removal is often limited by tumor infiltration along nerve plexuses, and residual tumors may undergo postsurgical tissue remodeling, producing localized regions of stiffened extracellular matrix (ECM). The impact of ECM stiffness on pNF1 growth and drug responses remains unclear. Using immortalized patient-derived pNF1 tumor cell lines cultured in 3D hydrogels with defined stiffness (1.5 kPa, soft; 7 kPa, stiff), we found that stiff ECM promoted spread morphology, increased growth, and progressive intracellular softening. Stiff ECM also reduced lysyl oxidase (LOX) expression, suggesting mechanoadaptive ECM remodeling, and increased P-glycoprotein expression. Under the same conditions, stiff ECM was associated with reduced sensitivity to selumetinib. These results provide the first evidence that ECM stiffening, including that plausibly associated with postsurgical remodeling, may contribute to pNF1 growth and reduced sensitivity to selumetinib in this 3D pNF1 culture model. Our findings highlight mechanobiology as a key regulator of tumor behavior and support further investigation of ECM-targeted strategies to improve outcomes in neurofibromatosis type 1 (NF1).

## 1. Introduction

Plexiform neurofibromas (pNF1s) are benign, diffuse peripheral nerve sheath tumors that arise in individuals with neurofibromatosis type I (NF1) and are driven by loss of neurofibromin, a key negative regulator of RAS/mitogen-activated protein kinase (MAPK) signaling [1,2,3,4,5,6]. These tumors grow diffusely along nerve plexuses and infiltrate surrounding soft tissues, often reaching substantial size and causing pain, functional impairment, and disfigurement [1,2,3,4,5,6]. Complete surgical removal is frequently difficult because of their infiltrative growth along nerves, blood vessels, and other critical organs, leading to residual tumor tissue and the need for repeated surgical interventions [1,2,3,4,5,6]. Although histologically benign, approximately 8–13% of pNF1s progress to malignant peripheral nerve sheath tumors (MPNSTs), which are aggressive and carry poor prognoses [7]. FDA-approved MEK inhibitors, selumetinib (AZD6244) [8] and mirdametinib (PD0325901) [9,10], can reduce tumor volume by ~30% and are used for inoperable, progressive, or symptomatic tumors, particularly when resection is incomplete or associated with high morbidity [11,12]. However, therapeutic responses are partial, and residual tumor regions may form mechanically distinct microenvironments that influence cellular signaling and drug efficacy. As a result, surgical resection remains the primary treatment when feasible, providing immediate tumor debulking and symptom relief [13,14]. However, because of their infiltrative growth and involvement with nerves, blood vessels, and other vital structures, pNF1s often persist or recur after incomplete surgical resection, necessitating repeated surgeries over a patient’s lifetime [15,16,17].

Our prior studies [18,19] demonstrated that fibroblast-derived secretome enhances pNF1 tumor cell growth, infiltrative behavior, and drug resistance in 3D cultures. In patient tumors, tumor cells comprise only ~6% of total volume, whereas fibroblasts represent ~60% and are the primary collagen producers [20]. Moreover, fibroblasts are more resistant to MEK inhibitors than tumor cells in 3D [19]. Based on these observations, it is plausible that fibroblasts in residual tumors—after surgery or drug treatment—could contribute to localized ECM stiffening. Such mechanically rigid niches may promote tumor expansion, invasion-associated behavior, and reduced therapeutic sensitivity, providing a plausible link between stromal remodeling and pNF1 tumor growth and treatment resistance. In addition to stromal influences, material properties of the extracellular environment, including stiffness and topography, have been shown to regulate key Schwann cell behaviors such as proliferation and migration in NF1 models [21].

ECM stiffening is increasingly recognized as a critical regulator of tumor biology in other solid cancers [22,23,24]. Increased matrix rigidity can arise from enhanced deposition and crosslinking of collagen and other ECM components, often mediated by enzymes such as lysyl oxidase (LOX), and is frequently associated with tumor growth, fibrosis, or chronic tissue remodeling [24,25,26]. ECM stiffening can also occur during wound healing after surgery, when fibroblast activation and collagen deposition locally increase matrix rigidity [27,28,29,30,31]. In tumor mechanobiology, matrices in the ~1–2 kPa range are commonly used to model physiologically compliant tissues, whereas matrices in the ~5–10 kPa or higher range are frequently used to represent fibrotic or pathologically stiffened microenvironments [22,25,32]. Such stiffened ECM enhances cell proliferation, promotes invasive behavior, and reduces drug efficacy through mechanotransduction pathways involving integrins, focal adhesion kinase (FAK), and yes-associated protein (YAP)/transcriptional co-activator with PDZ-binding motif (TAZ) signaling [22,25,32]. In addition, ECM stiffening influences cytoskeletal organization and intracellular mechanics, creating a mechanically distinct microenvironment that feeds back to regulate tumor cell behavior [32,33,34]. Because residual pNF1 tumors often persist after surgery, it is plausible that postsurgical tissue remodeling—including localized ECM stiffening—occurs, although this has not been systematically characterized. Understanding how ECM stiffness regulates pNF1 growth, invasion, and drug responsiveness could uncover mechanisms contributing to tumor persistence and reduced therapeutic response, providing clinically relevant insights for NF1 patients. Accordingly, we selected 1.5 kPa and 7 kPa matrices to represent experimentally controlled soft vs. stiff ECM conditions within these commonly used ranges. Direct measurements of ECM stiffness in human pNF1 tissues, including postsurgical or fibrotic regions, remain limited; therefore, these values are intended to model biologically plausible mechanical conditions rather than exact in vivo measurements.

To address this knowledge gap, we developed a three-dimensional (3D)/4D (3D in real-time) hydrogel-based culture system in which immortalized patient-derived pNF1 tumor cells are embedded in matrices of precisely tuned stiffness. This system models ECM conditions ranging [24,35] from compliant tissue to fibrotic, stiffened regions, allowing controlled assessment of stiffness-dependent effects on tumor growth, ECM remodeling, and drug response. Understanding ECM stiffness is particularly important in pNF1s, as surgical resection—the mainstay treatment [13,14]—often leaves residual tumors, and postsurgical remodeling can locally stiffen the matrix. In pNF1s, neoplastic tumor cells arise from Schwann cell (SC)–derived lineages with biallelic NF1 loss (*NF1^−/−^*), whereas surrounding stromal and non-neoplastic SCs are predominantly *NF1^+/−^*. Accordingly, this study focuses specifically on the mechanoadaptive responses of *NF1*-deficient tumor cells to pathologically stiffened ECM conditions, rather than comparing baseline mechanosensitivity between tumor and normal SCs. Our platform thus enables investigation of how mechanically altered microenvironments in residual tumors may drive tumor growth, infiltrative behavior, and reduced therapeutic sensitivity, providing a mechanistic framework for mechano-aware strategies in NF1.

## 2. Materials and Methods

### 2.1. Reagents and Antibodies

LunaX™ PureMatrix–photocrosslinkable ECM with low or high stiffness and LunaX™ crosslinker were purchased from Gelomics (Kelvin Grove, Queensland, Australia). Phenol red-free Dulbecco’s Modified Eagle Medium (DMEM) and MycoZap^TM^ Plus-CL were purchased from Lonza (Basel, Switzerland). Fetal bovine serum (FBS) was obtained from Cytiva (Marlborough, MA, USA). Selumetinib (AZD6244) was purchased from Selleckchem (Houston, TX, USA). L-glutamine and all other chemicals, unless otherwise specified, were purchased from Sigma-Aldrich (St. Louis, MO, USA). LIVE/DEAD^TM^ Viability/Cytotoxicity kit, FITC-phalloidin, Hoechst33342 and 4′-6-Diamidino-2-phenylindole (DAPI) were purchased from ThermoFisher Scientific (Waltham, MA, USA). Antibodies against lysyl oxidase (LOX) and p-glycoprotein (Pgp) were purchased from Abcam (Cambridge, UK).

### 2.2. Cell Lines and Cell Maintenance

Two immortalized human plexiform neurofibroma cell lines (*NF1*^−/−^; hereafter referred to as pNF1 tumor cells), ipNF95.11bC and ipNF05.5, were obtained from ATCC (Manassas, VA, USA) and previously described [18,19,36]. Cells were maintained as monolayers in DMEM/high glucose supplemented with 10% FBS at 37 °C, 5% CO_2_. Routine RT-PCR screening confirmed the absence of mycoplasma contamination.

### 2.3. Three-Dimensional (3D)/4D (3D in Real-Time) Culture Models

3D cultures were established in our patented microfluidic culture devices called TAME (tissue architecture and microenvironment engineering; Patent#, US10227556B2) chips [37] as previously published [18,19]. Detailed fabrication of TAME chips, including separate and linked well designs [37], and protocols for 3D cultures have been described previously [18,19]. Embedding of cells in LunaX™ PureMatrix–photocrosslinkable ECM, was performed according to the manufacturer’s instructions [38,39,40] to generate 3D hydrogels with defined stiffness values of 1.5 kPa (soft) and 7 kPa (stiff). According to manufacturer-provided stiffness–crosslinking relationships, low-stiffness matrices generated at ~50 s correspond to ~1.5 kPa, while high-stiffness matrices under similar exposure conditions reach approximately 7.5 (range, 7–9) kPa, reflecting the higher polymer density of the formulation. These stiffness values were selected to represent commonly used soft vs. stiff conditions in 3D tumor mechanobiology models [22,24,41,42]. These conditions were generated within the same hydrogel system, minimizing differences in matrix composition while varying mechanical properties. Briefly, 50,000 cells were mixed with 50 mL of 3D hydrogel with low or high stiffness and 50 mL of photoinitiator, plated in the glass-bottom wells of TAME chips and exposed to LunaX™ crosslinker for 50 s. The cultures were then maintained in DMEM supplemented with 5% FBS for the indicated periods (3–10 days). Prior to live-cell 3D imaging, nuclei were stained with Hoechst 33342 to assess cell integrity and morphology. Total live-cell numbers were quantified by counting intact, healthy nuclei that co-localized with viability markers (e.g., Calcein-AM).

### 2.4. Brillouin Microscopy

The mechanical properties of cells were quantified using a confocal Brillouin microscope. The detailed configuration of this microscope can be found in a previous publication [43]. Briefly, a 660 nm continuous-wave laser was used as the light source. The laser beam was coupled into a commercial optical microscope (IX83; Olympus, Tokyo, Japan) and focused into the sample using an objective lens (0.6 NA, Olympus), yielding a beam spot of 0.7 × 0.7 × 2.5 µm. The back-scattered Brillouin signal was collected by the same objective and sent to a Brillouin spectrometer for analysis. During experiments, 10–20 mW laser power was applied to each cell, and Brillouin images of the cell were collected with an acquisition speed of 50 ms per pixel and a step size of 1 µm. The Brillouin shifts of all pixels were then averaged to represent the stiffness of each cell. The Brillouin shifts of the surrounding gels were also recorded during each experiment to quantify their mechanical stability.

The confocal Brillouin microscopy is based on the optical process known as spontaneous Brillouin scattering, in which the scattered light experiences an optical frequency shift, called Brillouin shift, due to the interaction of the incident light and the material. In backward-scattering configuration, the elastic longitudinal modulus $M^{\prime }$ is related to the Brillouin shift $\omega_{B}$ by

$$\omega_{B}=\frac{2n}{\lambda }\sqrt{\frac{M^{\prime }}{\rho }}sin\left(\frac{\theta }{2}\right)$$

where λ is the wavelength of incident light, n and ρ are refractive index and density of the material. For some biological materials such as cells, the ratio of $n/\sqrt{\rho }$ can be approximated as constant [44]. Therefore, the measured Brillouin shift can be used to estimate the relative change of mechanical modules.

### 2.5. Drug Treatment

Selumetinib (10 µM) was selected based on our previous 3D culture studies in immortalized patient-derived pNF1 tumor cell lines, in which this concentration produced consistent and reproducible functional responses, including partial inhibition of cell growth/viability and increased proteolysis under prolonged treatment conditions [18,19,45]. Selumetinib treatment was initiated 24 h after embedding cells in 3D hydrogels, allowing initial establishment of multicellular tumor structures prior to drug exposure.

### 2.6. Live-Cell LIVE/DEAD^TM^ Viability/Cytotoxicity Assay

This assay was performed as described in our previous studies [19,46], and is summarized briefly here. Three-dimensional cultures were incubated with Calcein AM (live cells, green) for 30 min at 37 °C, followed by a single wash with warm PBS. Fresh warm culture media were then added according to the manufacturer’s instructions and previously published protocols [18,19]. Prior to live-cell imaging, nuclei were counterstained with Hoechst 33342 to confirm nuclear morphology and viability. Live-cell imaging was performed using confocal microscopy under physiological conditions. Quantification of viable cells was performed in 3D using Volocity software (Version 7.0.0; PerkinElmer, Waltham, MA, USA) by counting intact nuclei colocalized with Calcein AM-positive cytoplasm, excluding fragmented or condensed nuclei.

### 2.7. Image Acquisition for Quantitative Analysis in 3D

Live-cell imaging and quantitative analysis were performed in 3D as previously described [18,19,37]. Briefly, optical sections spanning the full depth of the 3D cultures were acquired from four contiguous fields (2 × 2 or 3 × 3) using either an upright Zeiss LSM 780 confocal microscope (Carl Zeiss, Jena, Germany) equipped with a temperature- and CO_2_-controlled environmental chamber, or an inverted Leica Stellaris 5 system (Leica Microsystems, Deerfield, IL, USA) with a Tokai Hit stage-top incubator to maintain physiological conditions. Image stacks were reconstructed and analyzed in 3D using Volocity software to visualize and quantify viable cell distributions. In reconstructed volumes, spatial orientation is indicated by arrows showing the *x*-axis (green), *y*-axis (red), and *z*-axis (blue) in the lower corner of each image.

### 2.8. Immunofluorescence Staining

pNF1 tumor cells were cultured for 5 days in 3D hydrogels of either soft or stiff ECM. Following culture, cells were fixed with 10% formaldehyde for 10 min at room temperature (RT), washed with PBS, and permeabilized for 5 min using 0.2% Triton X-100 in PBS. Cells were then blocked with 1% BSA for 1 h at RT and incubated with primary antibodies against LOX or Pgp for 3 h at RT. After washing, cells were incubated with appropriate fluorescent secondary antibodies for 1 h at RT. Nuclei were counterstained with DAPI. Images were acquired using an inverted Leica Stellaris 5 confocal microscope (Leica Microsystems, Deerfield, IL, USA). Quantification of LOX and Pgp fluorescence intensity was performed using ImageJ 1.54g software (National Institutes of Health, Bethesda, MD, USA).

### 2.9. Statistical Analysis

Data are presented as bar graphs or box-and-whisker plots. In bar graphs, bars represent the mean ± standard deviation (SD). In box-and-whisker plots, boxes represent interquartile ranges and whiskers minimum and maximum values. The number of replicates is provided in each corresponding figure legend. Statistical significance was assessed using either one-way ANOVA followed by Tukey’s post hoc test for multiple group comparisons, or Student’s *t*-test for comparisons between two groups. A *p*-value ≤ 0.05 was considered statistically significant throughout the study.

## 3. Results

### 3.1. Stiff ECM Promotes Spread Morphology and Enhances 3D Growth of pNF1 Tumor Cells

Because pNF1s often persist or recur after incomplete surgical resection [15,16,17] and postsurgical remodeling may stiffen the ECM [27,28,29,30,31], we first examined how matrix stiffness affects tumor cell behavior. Two patient-derived immortalized pNF1 cell lines, ipNF95.11bC and ipNF05.5, were cultured in 3D hydrogels with defined stiffness values of 1.5 kPa (soft) and 7 kPa (stiff). These stiffness values were selected based on prior mechanobiology studies in multiple tumor models [24,35], where matrices in the ~1–2 kPa range are commonly used to represent physiologically soft tissues, whereas matrices in the ~5–10 kPa range are widely employed to model fibrotic or mechanically stiffened tumor microenvironments, allowing us to model both compliant and fibrotic tumor conditions.

**Figure 1:**
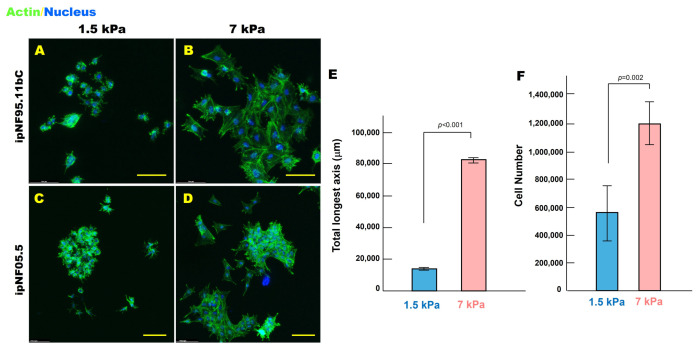
**pNF1 tumor structures exhibit a more spread morphology and increased growth in stiff ECM compared with soft ECM in 3D culture.** (**A**–**D**) *En face* views of 3D reconstructions of pNF1 structures formed by ipNF95.11bC (**A**,**B**) and ipNF05.5 (**C**,**D**) monocultures grown in soft (1.5 kPa) or stiff (7 kPa) ECM for 10 days. Actin fibers (green, phalloidin) and nuclei (blue) are shown. Scale bars: 70 µm (top row) and 115 µm (bottom row). (**E**,**F**) Quantification of structure size (longest axis; (**E**)) and cell number (**F**) of 3D ipNF95.11bC cultures measured in 3D using Volocity. Data represent mean ± SD (*n* = 4).

After 10 days, cells were stained with phalloidin to visualize filamentous actin and counterstained with Hoechst 33342 for nuclei. Three-dimensional reconstruction in Volocity revealed that cells in soft ECM maintained a compact and rounded morphology, whereas cells in stiff ECM adopted a more spread and expanded shape, reflected by a significant increase in the longest axis length in 3D (Figure 1A–D). Volumetric reconstruction further demonstrated that structure size and total cell number were significantly higher in stiff ECM than in soft ECM for 3D ipNF95.11bC structures (*p* < 0.01) (Figure 1E,F), indicating that increased ECM stiffness can drive both morphological expansion and enhanced tumor growth in 3D pNF1 culture conditions.

### 3.2. Stiff ECM Induces Progressive Intracellular Softening in 3D pNF1 Tumor Structures

**Figure 2:**
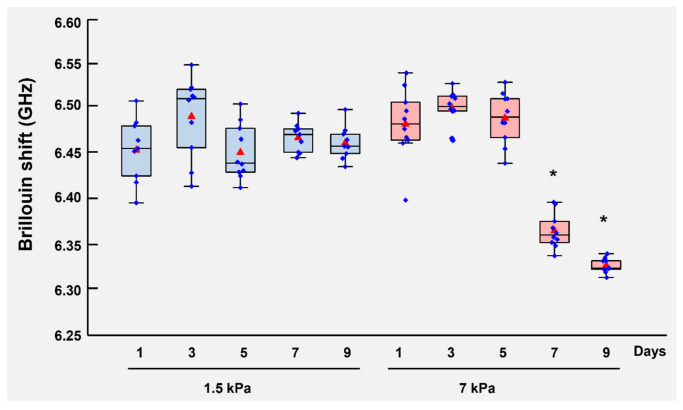
**Stiff ECM induces progressive intracellular softening in 3D pNF1 tumor structures.** Immortalized pNF1 tumor cells, ipNF95.11bC, were embedded in soft (1.5 kPa) or stiff (7 kPa) 3D ECM hydrogels and cultured for 9 days. Intracellular cytosolic stiffness was quantified at the indicated times using Brillouin microscopy. Individual cell bodies were scanned longitudinally over time to assess stiffness adaptations in response to ECM mechanics. The horizontal line within each box represents the median, red triangles indicate the mean values, blue dots represent individual measurements, boxes indicate the interquartile range, and whiskers represent the data range. * *p* < 0.01 *vs*. Day 1 (*n* = 3).

**Figure A1:**
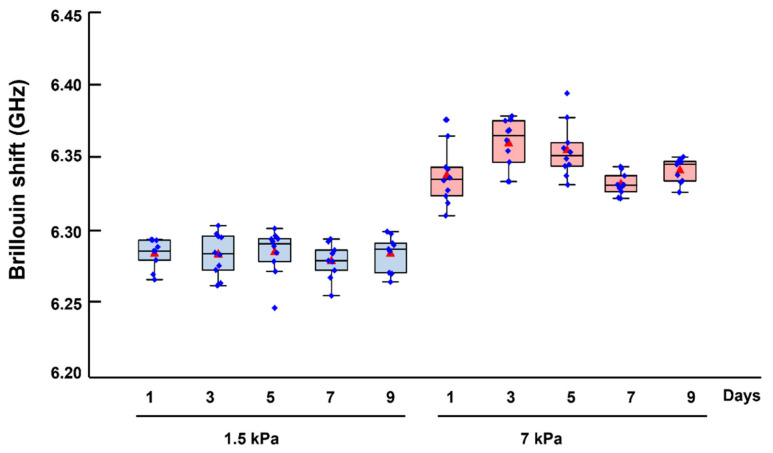
Brillouin microscopy measurements of ECM confirm stable and distinct mechanical properties of soft and stiff hydrogels. Brillouin microscopy was used to measure ECM (hydrogel) regions in the same samples used for intracellular measurements (Figure 2). The horizontal line within each box represents the median, red triangles indicate the mean values, blue dots represent individual measurements, boxes indicate the interquartile range, and whiskers represent the data range. Soft (1.5 kPa) and stiff (7 kPa) hydrogels showed distinct Brillouin shifts that remained stable over the culture period, confirming maintained mechanical differences between conditions (*n* = 3).

Because decreased cellular stiffness has been linked to enhanced deformability and infiltrative behavior in multiple solid tumors [42,47,48,49], we next measured intracellular stiffness in our 3D pNF1 cultures to assess whether ECM mechanical cues similarly affect infiltrative potential. Intracellular stiffness was quantified using Brillouin microscopy [50,51], a non-invasive, high-resolution technique that enables real-time, quantitative measurement of cytosolic mechanics in living 3D cultures without disrupting tissue architecture. Cells were cultured in soft (1.5 kPa) or stiff (7 kPa) ECM for 9 days. Cells in soft ECM maintained relatively stable intracellular stiffness throughout the culture period (the average Brillouin shifts were within the range of 6.44 GHz and 6.48 GHz) whereas cells in stiff ECM exhibited progressive cytosolic softening over time (the average Brillouin shift dropped from 6.50 GHz on Day 7 to 6.32 GHz on Day 9) (Figure 2). To further validate the mechanical distinction between ECM conditions, Brillouin microscopy measurements of the ECM (hydrogel) were acquired as control regions alongside intracellular measurements in the same samples (Figure A1). The 1.5 kPa and 7 kPa hydrogels showed distinct Brillouin shifts (~6.25–6.30 GHz and ~6.30–6.40 GHz, respectively), indicating a consistent stiffness-dependent trend within this system and supporting that the two ECM conditions remain mechanically distinct under the experimental conditions. These findings demonstrate that stiff ECM induces a time-dependent reduction in intracellular stiffness, consistent with increased cellular deformability. This stiffness-dependent intracellular adaptation suggests that mechanical cues from the ECM may promote increased cellular deformability, potentially facilitating increased interactions with and adaptation to surrounding matrix regions. Together, these results indicate that ECM stiffness not only regulates tumor growth but also regulates cellular mechanical properties associated with invasion-related phenotypes.

### 3.3. ECM Stiffness Regulates Lysyl Oxidase (LOX)-Associated Matrix Remodeling

**Figure 3:**
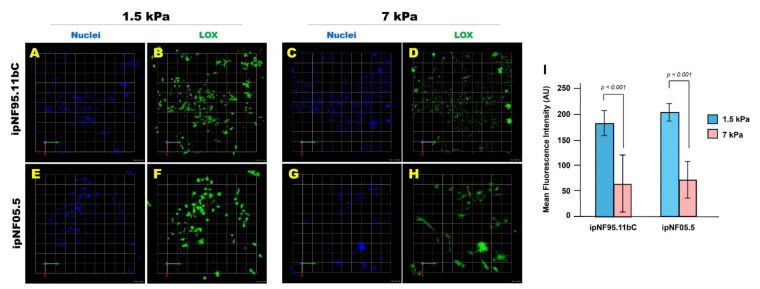
**Stiff ECM downregulates lysyl oxidase (LOX) expression in 3D pNF1 tumor structures.** (**A**–**H**) *En face* views of 3D reconstructions of pNF1 tumor structures formed by ipNF95.11bC (**A**–**D**) and ipNF05.5 (**E**–**H**) cultured in soft (1.5 kPa) or stiff (7 kPa) 3D ECM for 10 days. LOX expression is shown in green, and nuclei are labeled in blue. Each grid square corresponds to 110 µm (**A**–**D**) or 58 µm (**E**–**H**). (**I**) Quantification of LOX fluorescence intensity was performed using ImageJ. Data represent mean ± SD (*n* = 3).

Because intracellular mechanical adaptation may influence interactions with the surrounding matrix, we measured LOX expression, an enzyme that crosslinks collagen and elastin to regulate ECM stiffness [24,25,26]. pNF1 tumor cells were cultured for 10 days in soft or stiff ECM, and LOX expression was quantified by immunofluorescence. Tumor structures in soft ECM expressed significantly higher levels of LOX than those in stiff ECM (Figure 3). This may reflect a mechanoadaptive response [52], although the underlying mechanism was not directly tested in this study. In this context, pNF1 tumor cells in compliant matrices may upregulate LOX, whereas cells in stiff matrices may show reduced LOX expression. Notably, this inverse relationship between ECM stiffness and LOX expression is consistent with the possibility that tumor cells dynamically adjust matrix-remodeling programs in response to their mechanical environment. Importantly, these findings align with our intracellular softening results: stiff ECM is associated with cytosolic softening while also showing reduced LOX expression, highlighting potentially coordinated mechanical adaptation at both cellular and extracellular levels.

### 3.4. Stiff ECM Enhances P-Glycoprotein (Pgp) Expression and Reduces Sensitivity to Selumetinib

Finally, to determine whether stiffness-driven intracellular and extracellular adaptations have functional consequences for therapy, we evaluated the effect of ECM stiffness on drug response. Mechanotransduction and ECM mechanics regulate the expression and activity of drug-efflux transporters [e.g., ATP-binding cassette subfamily B member (ABCB) 1/Pgp, ATP-binding cassette subfamily C member (ABCC) 1/multidrug resistance-associated protein (MRP) 1 and ATP-binding cassette subfamily G member (ABCG) 2/breast cancer resistance protein (BCRP)] via integrin–FAK/ILK and YAP/TAZ-dependent pathways, and this link has been demonstrated in multiple tumor models including breast, lung, colorectal and others [53,54,55,56], highlighting its clinical relevance for understanding partial responses to selumetinib in pNF1 patients.

**Figure 4:**
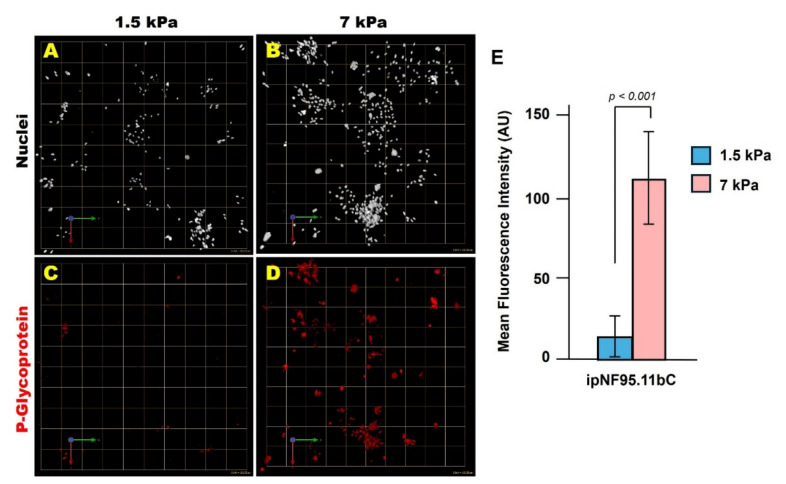
**Stiff ECM promotes P-glycoprotein expression in 3D pNF1 tumor structures.** (**A**–**D**) *En face* views of 3D reconstructions of pNF1 tumor structures formed by ipNF95.11bC cultured in soft (1.5 kPa) or stiff (7 kPa) 3D ECM for 10 days. P-glycoprotein (Pgp) expression is shown in red, and nuclei are labeled in pseudo-white. Each grid square corresponds to 110 µm. (**E**) Quantification of Pgp fluorescence intensity was performed using ImageJ. Data represent mean ± SD (*n* = 3).

**Figure 5:**
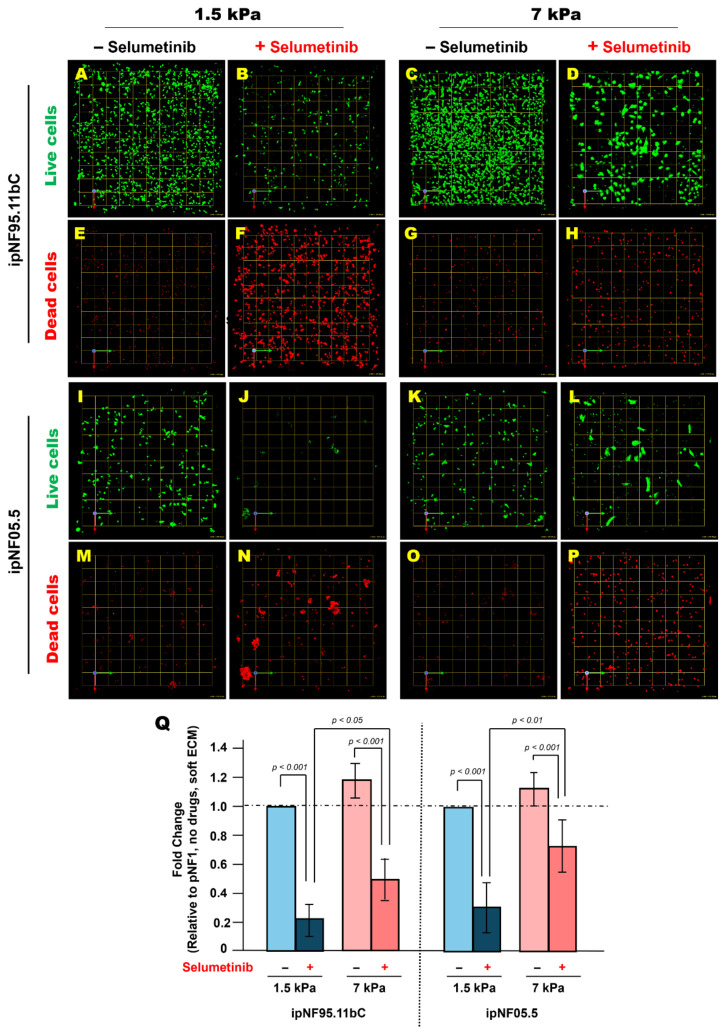
**Stiff ECM reduces sensitivity to selumeinib in 3D pNF1 tumor structures after 5-day treatment.** (**A**–**P**) *En face* views of 3D reconstructions of pNF1 tumor structures formed by ipNF95.11bC (**A**–**H**) and ipNF05.5 (**I**–**P**) in monocultures without treatment (first and third panels) or after 5-day treatment with 10 μM selumetinib (second and fourth panels). Live and dead tumor cells were stained with Calcein-AM (green) and ethidium homodimer-1 (red), respectively. Images are tiled from 4 contiguous fields. Grid, 174 µm. (**Q**) Quantification of total live pNF1 tumor cells in 3D monocultures using Volocity. Data are expressed as fold change relative to untreated cells within each ECM condition. Each bar represents mean ± SD (*n* = 3).

Tumor cells were cultured for 10 days in soft or stiff ECM and analyzed for Pgp expression by immunofluorescence. Tumor structures in stiff ECM exhibited significantly higher Pgp levels than those in soft ECM (Figure 4). To assess whether this stiffness-associated upregulation of Pgp expression may affect therapeutic response, tumor cells were treated with 10 mM selumetinib for 5 days, starting 24 h after embedding in 3D hydrogels. Selumetinib was selected as a clinically relevant MEK inhibitor for pNF1 and because its response profile and working concentration have been well characterized in our prior 3D culture studies [18,19]. Live and dead cells were labeled with Calcein-AM and ethidium homodimer-1, respectively, and total cell viability was quantified using Volocity. Tumor structures in stiff ECM retained substantially more viable cells after treatment compared with those in soft ECM, indicating reduced sensitivity to selumetinib under stiff ECM conditions (Figure 5). To facilitate direct comparison across stiffness conditions, relative changes in cell viability (drug-treated *vs*. untreated within each ECM condition) were calculated. This analysis revealed a greater fold reduction in viability in soft ECM (1.5 kPa) compared with stiff ECM (7 kPa), further highlighting stiffness-dependent differences in drug sensitivity (Figure 5Q). While these findings are consistent with stiffness-dependent effects, we cannot exclude the possibility that associated changes in hydrogel properties (e.g., diffusion or matrix architecture) may also contribute. These findings demonstrate that ECM stiffness is associated with increased expression of Pgp protein expression and reduced response to selumetinib, suggesting that the mechanical microenvironment may contribute to therapeutic variability in pNF1 tumor cells.

Collectively, these results demonstrate that stiff ECM is associated with multiple pNF1 phenotypes, including growth, spread morphology, intracellular softening, reduced LOX expression, and decreased sensitivity to selumetinib. Together, these coordinated changes highlight the multifaceted role of mechanical cues in regulating tumor cell behavior. This integrated response underscores the potential role of ECM stiffness in modulating tumor behavior and selumetinib response in pNF1s.

## 4. Discussion

This study demonstrates that ECM stiffness is a critical, previously underappreciated determinant of pNF1 tumor cell behavior. Using a tunable 3D hydrogel system combined with quantitative mechanophenotyping, we show that mechanically distinct ECM conditions within the same hydrogel system are associated with changes in multiple dimensions of pNF1 tumor behavior, including morphology, intracellular mechanics, ECM remodeling, and response to targeted therapy. These findings expand the framework of pNF1 biology beyond canonical RAS/MAPK hyperactivation and identify ECM stiffness as a potential contributor to residual tumor growth and therapy response.

A major and novel observation is that stiff ECM promotes spread morphology and enhances growth of pNF1 tumor structures. Clinically, this is particularly relevant because surgical resection remains the primary treatment for pNF1s [13,14], yet complete removal is limited by the diffuse and infiltrative nature of these tumors [15,16,17]. Residual tumor tissue may reside within mechanically rigid, fibrotic niches [27,28,29,30,31], which could plausibly favor regrowth and expansion. However, the stiffness values used in this study are based on ranges commonly applied in tumor mechanobiology models [22,24,41,42] and do not represent direct measurements from pNF1 tissues; therefore, the relevance to postsurgical or fibrotic conditions should be considered plausible but not directly validated. Future studies incorporating direct mechanical measurements of pNF1 tissues will be important to further define clinically relevant stiffness ranges.

At the cellular level, stiff or stiffening ECM environments can elicit mechanoadaptive responses in which tumor cells become more deformable or exhibit reduced intracellular stiffness [57,58]. Our findings with pNF1 tumor cells align with this behavior. Such decreased stiffness has been consistently associated with enhanced deformability and greater invasion-associated potential across multiple solid tumor models [42,47,48,49,59]. Although pNF1s are histologically benign, they grow diffusely along nerve pathways [1,2,3,4,5,6], and our data suggest that stiffness-induced cytoskeletal softening may reflect cytoskeletal remodeling and adaptive changes in cell mechanics in response to a mechanically restrictive microenvironment. In this context, intracellular softening may represent a mechanoadaptive phenotype in which cells embedded within a stiff matrix become more deformable, potentially facilitating their ability to accommodate mechanical constraints and interact with surrounding matrix structures.

ECM rigidity also altered expression of LOX, a key collagen- and elastin-crosslinking enzyme [24,25,26]. We observed higher LOX expression in soft ECM and reduced LOX in stiff ECM, a finding that does not align with previously reported association of LOX with matrix stiffening [24,25,26]. One possible explanation is that reduced LOX expression in stiff ECM represents a compensatory or mechanoadaptive response. In this context, cells embedded within an already stiffened environment may reduce further LOX-mediated matrix reinforcement. This pattern may be consistent with mechano-responsive feedback loops described in other tissues and tumor systems [52]. These findings indicate coordinated intracellular and extracellular adaptation, in which pNF1 tumor cells remodel both their cytoskeleton and matrix-regulatory programs in response to ECM stiffness. Together with intracellular softening, these observations are consistent with coordinated mechanoadaptive responses to ECM stiffness, although the causal relationships remain to be defined.

Although intracellular softening has been linked to increased deformability and invasive behavior in other tumor systems [42,47,48,49], the present study does not directly test whether this phenotype contributes to enhanced growth, infiltrative behavior, or reduced selumetinib sensitivity in pNF1 tumor cells. Notably, while intracellular softening becomes evident during the culture period, this early mechanical adaptation alone may not fully account for the reduced drug sensitivity observed at later time points. Instead, prolonged exposure to stiff ECM likely promotes additional downstream changes, including altered ECM remodeling and drug response-associated adaptations, which together contribute to the observed therapeutic response. Changes in cell mechanical state may also influence signaling pathways or drug accessibility; however, these mechanisms were not directly examined in the present study.

In the present study, invasion was not directly measured using cell tracking, migration distance, or matrix infiltration assays. Instead, we quantified spread morphology and structural expansion of tumor spheroids in 3D. While these features are often associated with invasive behavior, they do not directly represent cell migration or invasion. Therefore, the observed phenotypes should be interpreted as invasion-associated rather than definitive measures of invasion. Future studies incorporating single-cell tracking, directional motility analysis, or matrix infiltration assays will be important to distinguish true invasion from proliferative spreading under stiffness-controlled conditions.

Modulation of hydrogel stiffness can be accompanied by changes in other material properties, including porosity, diffusion characteristics, ligand presentation, and matrix architecture [60]. In addition, material cues independent of stiffness, such as topography, have been shown to influence Schwann cell behavior in NF1 models [21], further highlighting the complexity of isolating stiffness-specific effects. Although we used the same hydrogel system to minimize compositional differences, these parameters were not directly measured in the present study. Therefore, while our data are consistent with stiffness-dependent effects, we cannot exclude the possibility that associated material properties contribute to the observed phenotypes, including differences in drug response.

Stiff ECM also increased Pgp expression and was associated with reduced sensitivity to selumetinib, suggesting that mechanical cues in the tumor microenvironment may influence drug response. Notably, our prior study [19] demonstrated that fibroblast-derived secretome upregulates Pgp expression and can functionally mediate drug resistance in pNF1 tumor cells. Together, these observations suggest that both stromal signals from fibroblasts and mechanically stiffened ECM may converge on Pgp-associated pathways that regulate therapeutic sensitivity. However, in the present study, Pgp was assessed at the protein level only and functional efflux assays and transcript-level analyses were not performed. Future studies will be needed to determine whether increased Pgp expression directly contributes to reduced selumetinib sensitivity observed under stiff ECM conditions.

Given that surgical resection remains the primary treatment for pNF1s [13,14] and clinical responses to MEK inhibition are often partial [11,12], these findings underscore the translational relevance of considering ECM mechanics and stromal contributions in evaluating residual tumor behavior and optimizing therapy. Consistent with the primarily cytostatic mechanism of MEK inhibition, the observed reduction in viable cell number after prolonged treatment likely reflects cumulative effects on tumor cell growth rather than nonspecific drug toxicity.

The 3D hydrogel-based model represents a major methodological advance for pNF1 research. By enabling precise tuning of ECM stiffness while preserving native 3D architecture, this platform allows controlled assessment of stiffness-dependent growth, cytoskeletal mechanics, ECM remodeling, and drug response. Building on our prior 3D coculture work incorporating bioengineered neuronal axons [46], this system demonstrates versatility and can be further adapted to study additional microenvironmental interactions, such as tumor–axon crosstalk, alongside mechanobiology. Importantly, it provides a physiologically relevant framework for preclinical testing of mechanotransduction-targeted therapies, LOX inhibitors, or antifibrotic approaches, and may facilitate incorporation of mechanical biomarkers into clinical studies. These findings also have important implications for future research and potential clinical translation. Future studies should investigate the molecular pathways linking ECM stiffness to intracellular softening, ECM remodeling, and drug response, including mechanotransduction signaling involving integrins, FAK, and YAP/TAZ [22,25,32]. In addition, functional validation of drug resistance mechanisms, such as the role of P-glycoprotein, will be important to establish causal links between mechanical cues and therapeutic outcomes. From a translational perspective, our results suggest that targeting ECM remodeling or mechanical properties of the tumor microenvironment—such as through LOX inhibition, antifibrotic strategies, or modulation of matrix stiffness—may enhance the efficacy of MEK inhibitor therapies. Incorporating mechanical features of the tumor microenvironment into preclinical models and clinical evaluation may therefore improve therapeutic response and long-term disease control in pNF1s.

The cell models used in this study are immortalized patient-derived human pNF1 cell lines rather than primary tumor cells. While these models provide a controlled and reproducible system to interrogate tumor cell–intrinsic responses to ECM mechanics, they do not fully recapitulate the cellular heterogeneity and microenvironmental complexity of pNF1s in vivo, including contributions from fibroblasts, immune cells, vascular components, and nerve-associated elements. Accordingly, the findings presented here should be interpreted within the context of a simplified 3D monoculture system.

Several additional limitations should be acknowledged. The absence of normal human SCs or *NF1^+/−^* SCs as comparative controls limits assessment of baseline mechanosensitivity and tumor-specific vs. lineage-dependent responses. Only two immortalized patient-derived pNF1 cell lines were evaluated, and expansion to additional models and anatomical tumor sites will be important to capture biological heterogeneity. Finally, the mechanotransduction pathways linking ECM stiffness to intracellular softening, LOX regulation, and reduced sensitivity to selumetinib remain to be defined. Future studies should evaluate whether ECM stiffness similarly regulates responses to other MEK inhibitors, including mirdametinib, and will benefit from incorporating multicellular systems, organoids, explants, or in vivo models to validate and extend these findings.

## 5. Conclusions

This work identifies ECM stiffness as a central regulator of pNF1 tumor cell behavior, with direct implications for growth, invasion-associated phenotypes, matrix remodeling, and response to selumetinib in this model. Understanding stiffness-dependent tumor behavior and drug response may be relevant in the context of surgery, where residual tumors could plausibly experience postsurgical ECM stiffening. The study provides a mechanistic framework and a versatile 3D hydrogel platform to investigate pNF1 biology and guide the development of mechano-aware therapeutic strategies, with clear clinical and translational significance.

## 6. Patents

The present study is associated with the U.S. Patent US10227556B2.

## Data Availability

The original contributions presented in this study are included in the article. Further inquiries can be directed to the corresponding authors.
